# Confrontation with others' emotions changes bodily resonance differently in those with low and high levels of depersonalization

**DOI:** 10.1098/rstb.2023.0248

**Published:** 2024-08-26

**Authors:** Helge Gillmeister, Ieva Šmate, Dimitra Savva, Haojie Li, Christina Parapadakis, Julia Adler

**Affiliations:** Department of Psychology, University of Essex, Wivenhoe Park, Colchester CO4 3SQ, UK

**Keywords:** emotions, somatosensory, mirroring, depersonalization

## Abstract

We present novel research on the cortical dynamics of atypical perceptual and emotional processing in people with symptoms of depersonalization–derealization disorder (DP-DR). We used electroencephalography (EEG)/event-related potentials (ERPs) to delineate the early perceptual mechanisms underlying emotional face recognition and mirror touch in adults with low and high levels of DP-DR symptoms (low-DP and high-DP groups). Face-sensitive visual N170 showed markedly less differentiation for emotional versus neutral face–voice stimuli in the high- than in the low-DP group. This effect was related to self-reported bodily symptoms like disembodiment. Emotional face–voice primes altered mirror touch at somatosensory cortical components P45 and P100 differently in the two groups. In the high-DP group, mirror touch occurred only when seeing touch after being confronted with angry face–voice primes. Mirror touch in the low-DP group, however, was unaffected by preceding emotions. Modulation of mirror touch following angry others was related to symptoms of self–other confusion. Results suggest that others’ negative emotions affect somatosensory processes in those with an altered sense of bodily self. Our findings are in line with the idea that disconnecting from one's body and self (core symptom of DP-DR) may be a defence mechanism to protect from the threat of negative feelings, which may be exacerbated through self–other confusion.

This article is part of the theme issue ‘Sensing and feeling: an integrative approach to sensory processing and emotional experience’.

## 1. Introduction

It is increasingly recognized that perceptual processing pathways intertwine with emotional ones to colour our subjective sense of self, and to allow us to successfully navigate our physical and social world. Our subjective sense of self is rooted in processes of our own bodily experiences and our resonance with others, which begins in infancy but requires continuous maintenance throughout life, and includes sensations, actions and emotions (e.g. [1–3]). Multisensory-motor contingencies, such as the experience of seeing and feeling your own body move [4,5], or the physical interactions that accompany emotionally attuned caregiver responses to infants [1,6], provide the basis for developing an authentic sense of self (e.g. [7]; see also [8]). As inherently social beings, humans' internal representations thus depend on their developmental experiences with others and, in turn, guide how we perceive ourselves and the external world.

Our resonance with others’ sensations, actions and emotions is an automatic process known as mirroring. For example, watching someone else bite into a lemon activates one's own internal representation of this action, which triggers the accompanying automatic salivary response and the phenomenology of tasting something sour. The same mirroring process (mapping an internal representation to the external world) takes place also when we observe bodily experiences like touch. Experimental paradigms involving mirroring can effectively capture psychological phenomena of bodily self-awareness, including higher-level explicit functions such as body ownership, self-location and agency (for reviews see [9,10]), but also a more implicit basic sense of bodily self [11–14] and motor contagion (e.g. [15–17]).

Mirroring also takes place when we observe others' emotions, and is the basis of empathetic processes, as it allows us to resonate with others’ affective and cognitive states (e.g. [18–20]). In other words, emotional resonance may be seen as an automatic process that helps us to understand others and forms a basis for social interactions. However, to be effective agents in the (social) world, including for purposes of emotionally supporting others, the automatic matching of sensations, actions and emotions must be accompanied by self–other distinction—a physiological and psychological differentiation of inner (self) from outer (non-self) worlds (e.g. [21]).

Mirroring, therefore, plays a centrally important role both in developing bodily self-awareness and in understanding others. Here, we are interested in how mirroring processes are affected by being confronted with others' emotions. It has been proposed that others’ emotional facial expressions activate an observer's shared neural representations (mirroring), which give rise to automatic mimicry. The physiological feedback from this induces emotions (emotional contagion) that help the observer to better understand the other person (empathy) [22]. However, it is less clear how exposure to the affective states of others modulates the ongoing mapping between one's own and others’ *bodily* states.

Cardini *et al*. [23] showed that viewing touch on another person's face enhances the detection of simultaneous touches on one's own face, especially when the other person's face expresses fear (but not anger or happiness), because fearful faces signal threat in the environment. Interestingly, emotional enhancement of mirror touch for fearful faces was absent in those with higher levels of alexithymia [24], suggesting a link between the ability to process emotions and the mapping of others' bodily states onto one's own. To the best of our knowledge, the cortical temporal dynamics of these links are still unknown. Moreover, the question arises whether these processes differ, not just in those with difficulties identifying emotions (alexithymia), but for participants with an altered sense of bodily self (e.g. feelings of disembodiment). Disembodiment is present in depersonalization–derealization disorder (DP-DR), a psychological condition characterized by feelings of detachment from one's own self and body and/or from one's surroundings (e.g. [25–27]). For example, in this condition, one might have the experience of being an outside observer to one's own thoughts, feelings, sensations and body (depersonalization) or experience other people or objects as unreal, dream-like, lifeless or as if through a fog (derealization). In DP-DR, reality testing remains intact (e.g. [27]). DP-DR is typically accompanied by emotional numbness and can occur in healthy adults under conditions of stress or fatigue [27,28] or as a symptom of a mental disorder (e.g. panic disorder, post-traumatic stress disorder). When symptoms of DP-DR are persistent, they may indicate the presence of DP-DR, which causes clinically significant distress or impairments [29,30]. The prevalence of the disorder in the general population is around 1–2%, with both genders equally affected ([27,31,32]; see also [33]).

Previous studies have shown that DP-DR is associated with altered mirroring processes, especially for self-related stimuli [11,13,34]. An earlier study from our laboratory showed reduced tactile mirroring for touch occurring on an image of one's own face in those with high levels of DP-DR symptoms [11]. People with higher levels of DP-DR symptoms are also more susceptible to body ownership illusions (e.g. the rubber hand illusion; [35]), suggesting that feelings of disembodiment may also be associated with weaker self–other distinction, that is, more self–other confusion.

To test how mirroring of bodily sensations is modified by others' emotions, and how these processes might be altered in participants with DP-DR symptoms, we used electroencephalography/event-related potentials (EEG/ERPs) in a well established tactile mirroring paradigm [11,12,36–45]. In the current study, we modified this paradigm to measure the mirroring of touch on a viewed hand following the presentation of happy, angry or neutral faces paired with matching happy sounds (giggles), angry sounds (growls) and neutral sounds (uhh). We chose anger, rather than fear, to signal threat, because DP-DR has been associated with childhood emotional abuse and neglect [25,46,47]. The emotional stimuli were deliberately complex, consisting of both visual and auditory components, to enhance their realism and intensity while still allowing the measurement of ERPs to visual stimulus onsets. We measured differences in visual ERPs in resposne to angry, happy and neutral face–voice stimuli (N170; reflecting structural and emotional encoding of faces in occipitotemporal cortex; [48–50]) and in somatosensory ERPs to touch and no-touch stimuli (P45 and P100 components, reflecting bodily self-awareness in parietal cortex; e.g. [11,12,14,51]) in individuals with high and low levels of DP-DR symptoms. In addition to the Cambridge Depersonalization Scale (DPS), which measures DP-DR symptoms, participants completed self-report surveys on self–other confusion, anxiety, depression and interoceptive awareness.

Firstly, in participants without symptoms of DP-DR (low-DP group) we expected larger N170 amplitudes to emotional versus neutral face–voice stimuli, reflecting emotional processing [49,52], as well as larger P45 and P100 amplitudes to touch versus no-touch stimuli (‘mirror touch’; e.g. [12,14]). Further, we expected that mirror touch would be modulated by the emotional context of the preceding face–voice stimuli. Secondly and in contrast to the low-DP group, we expected that our high-DP group would show reduced emotional processing at N170, reflecting emotional numbness, as already indicated in an fMRI study of DP-DR patients [53]. We also expected them to show altered mirror touch, as already shown in our previous work [11]. Given the well documented emotional numbness and self-processing deficits experienced in DP-DR (e.g. [11,34,53]), we were particularly interested in the question of how emotional processing and mirror touch would interact in this group. Thirdly, given the heightened susceptibility to experience rubber-hand illusions in DP-DR [35], we hypothesized that our high-DP group would report more self–other confusion also in subjective measures taken from a psychodynamic tool (Operationalized Psychodynamic Diagnosis-Structure Questionnaire). Finally, we measured how the expected cortical emotional and mirror-touch effects were related to subjective measures on which we expected the groups to differ (disembodiment, emotional numbness, self–other confusion and interoceptive awareness).

## 2. Method

### (a) Participants

After initial screening via targeted or general University of Essex mailing channels of healthy adults with either low or high levels of depersonalization, a total of 50 individuals (21 low- and 29 high-DP individuals) participated in the EEG study. Table 1 (see Results) describes each sample group and shows group comparisons of demographics and other dependent variables. All participants had normal or corrected-to-normal vision, and none reported suffering from any mental disorder at the time. The study was carried out in line with ethical principles laid out in the Declaration of Helsinki, it obtained ethical approval from the University of Essex Science and Health Faculty ethics sub-committee, and participants gave informed written consent before taking part.

**Table 1.** Descriptive statistics of both depersonalization groups (*t*-tests for continuous variables and *χ*^2^- tests for categorical): including survey scores (MAIA, PHQ9, OPD-SQ, STICSA) and CDS scores. ***p ≤ 0.001. **p < 0.005. *p < 0.05. n.s., *p* > 0.05.

	low depersonalization group, CDS < 50 (*n* = 21) mean (s.d*.*)	high depersonalization group, CDS > 50 (*n* = 29) mean (s.d.)	group comparison
age (years)	22.9 (3.2)	24.5 (4.4)	*t*_48_ = 1.403, *p* = 0.167, *d* = 0.416; n.s.
sex	7 male; 31.82%	9 male; 31.03%	χ^2^_1_ = 0.03, *p* = 0.863; n.s.
handedness	19 right-handed; 86.36%	28 right-handed; 96.55%	χ^2^_1_ = 0.797, *p* = 0.372; n.s.
CDS	16 (11.9)	95 (41.6)	*t*_48_ = 8.434, *p* < 0.001; *d* = 2.581***
CDS (anomalous body experience)	2.3 (2.48)	20.8 (13.7)	*t*_48_ = 6.074, *p* < 0.001; *d* = 1.879***
CDS (emotional numbing)	3.5 (4.68)	22.7 (11.94)	*t*_48_ = 6.968, *p* < 0.001; *d* = 2.117***
STICSA somatic anxiety	1.5 (0.43)	1.8 (0.67)	*t*_48_ = 1.988, *p* = 0.053; *d* = 0.533; n.s.
STICSA cognitive anxiety	1.9 (0.80)	2.6 (0.63)	*t*_48_ = 3.274, *p* = 0.002; *d* = 0.972**
PHQ9	7.8 (6.83) (mild depression)	11.4 (4.59) (moderate depression)	*t*_48_ = 2.284, *p* = 0.027; *d* = 0.619*
OPD-SQ (self–other confusion)	1.4 (0.71)	2.2 (0.76)	*t*_44_ = 3.583, *p* = 0.001; *d* = 1.091***
MAIA noticing	2.95 (1.31)	2.91 (1.03)	*t*_48_ = 0.134, *p* = 0.894; *d* = 0.034; n.s.
MAIA not distracting	2.78 (0.89)	2.15 (0.96)	*t*_48_ = 2.768, *p* = 0.023; *d* = 0.681*
MAIA not worrying	2.56 (0.99)	2.38 (0.86)	*t*_48_ = 0.672, *p* = 0.505; *d* = 0.194; n.s.
MAIA attention regulation	2.77 (1.08)	2.43 (0.64)	*t*_48_ = 0.476, *p* = 0.636; *d* = 0.383; n.s.
MAIA self regulation	2.98 (1.29)	2.55 (1.08)	*t*_48_ = 1.273, *p* = 0.209; *d* = 0.361; n.s.
MAIA body listening	1.92 (1.29)	2.09 (1.45)	*t*_48_ = 0.430, *p* = 0.669; *d* = 0.124; n.s.
MAIA emotion awareness	2.94 (1.15)	3.13 (1.10)	*t*_48_ = 0.586, *p* = 0.561; *d* = 0.169; n.s.
MAIA trusting	3.08 (1.38)	2.59 (1.17)	*t*_48_ = 1.363, *p* = 0.179; *d* = 0.383; n.s.

### (b) Material and apparatus

To measure depersonalization levels, the Cambridge Depersonalization Scale (CDS) was used. It measures the self-rated frequency and duration of depersonalization symptoms experienced over the duration of the last six months [54] using 29 items regarding ‘strange and funny’ experiences. Items from the questionnaire regard the four dimensions of depersonalization [55]: anomalous body experience (e.g. ‘Parts of my body feel as if they didn't belong to me’), emotional numbing (e.g. ‘When I weep or laugh, I do not seem to feel any emotions at all’), anomalous subjective recall (e.g. ‘It seems as if things that I have recently done had taken place a long time ago’) and alienation from surroundings (e.g. ‘My surroundings feel detached or unreal, as if there was a veil between me and the outside world’). Using a Likert scale, participants rated frequency from 0 (never) to 4 (all the time) and duration from 1 (a few seconds) to 6 (more than a week) for each statement. All scores were added to compute each participant's global personalization score (0–290), with 70 being the clinical cut off for personalization disorder. In this study, those scoring less than 50 were included in the low-DP group; those with a score higher than 50 were in the high-DP group (see [11,13,35]).

Participants also completed a subscale measuring self–other confusion from the Operationalized Psychodynamic Diagnosis-Structure Questionnaire (OPD-SQ) [56], a measurement to assess personality dysfunction that has previously been related to dissociative symptoms in borderline personality disorder [57]. It includes seven statements describing different personal characteristics such as ‘Sometimes I'm afraid that the boundary between me and others will disappear’. Statements were rated on a 5-point Likert scale, ranging from ‘no agreement at all’ to ‘total agreement’. Higher OPD-SQ scores on this subscale indicate self–other confusion.

Additionally, participants filled in questionnaire items to measure depression (Patient Health Questionnaire-9; PHQ9) and anxiety (State–Trait Inventory for Cognitive and Somatic Anxiety; STICSA). This was aimed to allow us to match, where possible, anxiety and depression levels for participants to measure differences as a function of depersonalization without confounds from depression and anxiety, which are highly comorbid with depersonalization [58]. PHQ9 consists of nine questions (e.g. ‘poor appetite or over-eating’), and asks for frequency of events to be rated over the last 2 weeks on a 4-point Likert scale from 0 (not at all) to 3 (nearly every day) [59]. STICSA includes cognitive (related to anxious thoughts, like ‘I think the worst will happen’) and somatic (anxiety-related bodily feelings, such as ‘My face feels hot’) subscales, totalling 21 statements related to thoughts/feelings experienced ‘these days’ and asking participants to rate them on frequency using a 4-point Likert scale from 1 (not at all) to 4 (very much so) [60]. Higher scores on PHQ9 and STICSA are associated with higher depression and anxiety, respectively.

Finally, participants completed the Multidimensional Assessment of Interoceptive Awareness (MAIA) measure to assess eight different dimensions of subjective interoception [61]. It consists of 32 questions regarding how individuals notice, appraise and regulate interoceptive signals: noticing (for example, ‘I notice when I am uncomfortable in my body’), not-distracting, not-worrying, attention regulation, emotional awareness, self-regulation, body listening and trusting. Participants rated their experienced frequency for all statements on a Likert scale from 0 (never) to 5 (always). Higher scores indicate greater subjective interoceptive awareness.

**Figure 1.**
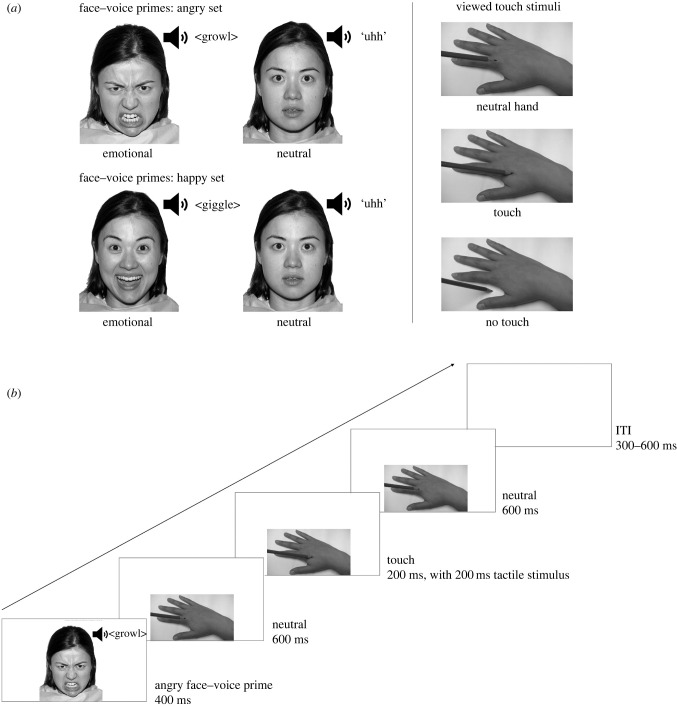
(*a*) Emotional and neutral face–voice prime stimuli (left panel) and viewed touch stimuli (right panel). One of the 18 different faces used in this study is shown, adapted with permission from Tottenham *et al*. [62]. One of the two different hand stimuli used in this study is shown. (*b*) Schematics of trial design showing an example trial (angry face–voice prime followed by a viewed touch stimulus). ITI, inter-trial interval.

In the experimental task, emotional primes included happy faces and voices (giggles), angry faces and voices (growls), and neutral faces and voices (‘uhh’ sounds). Angry, happy and neutral faces were obtained from the NimStim set of facial expressions [62], and angry, happy and neutral vocalizations were taken from the Montreal Affective Voices dataset [63] and shortened to 400 ms. There were nine different female and nine different male face models, paired with five different female and five different male vocalizations. Faces were presented close to life-size (17.1° horizontal × 22.6° vertical visual angle) in greyscale against a white background in the centre of a computer screen (figure 1*a*).

To probe tactile mirroring, we presented images of a hand with a pencil above it (neutral stimulus), of a hand being touched by a pencil in the region of the first dorsal interosseus muscle (touch stimulus) or of a hand with the pencil moved to the space next to it (no-touch stimulus). A male hand was shown to male participants, and a female hand was shown to female participants. Hands were presented close to life-size (20.7° horizontal × 11° vertical visual angle) in greyscale against a white background in the lower half of a computer screen (figure 1*a*). A tactile controller and mechanical solenoid stimulators (Heijo Research Electronics, London, UK) delivered suprathreshold taps by pushing a blunt plastic tip against the participant's skin whenever a current was passed through the solenoid. Taps were delivered to participants' left or right hands in the region of the first dorsal interosseus muscle at the same time during the presentation of each touch or no-touch image, such that felt and seen touches always occurred on the same hand (e.g. right). A long rectangular box covered by a piece of black cloth was placed on the table between the computer and participant to block participants’ view of their own hands, which were placed on their lap.

### (c) Procedure

Advertisements targeting participants aged between 18 and 45 years with potentially high and low levels of depersonalization, anxiety and/or depression were sent via three different University of Essex mailing channels. The emails included either the question ‘Do you sometimes suddenly feel as if you were not real, or cut off from the world?’ (depersonalization) or the questions ‘Do you often think that your friends are happier than you? Do you feel that you no longer enjoy things that used to give you pleasure? Do you often feel something in your chest without knowing why?’ (anxiety and depression) as well as the statement ‘If this feeling is completely unfamiliar to you, we would also like to hear from you, as we need additional participants for our comparison group’, followed by an invitation to the study. Before the EEG study, participants gave informed written consent and completed survey measures.

EEG was recorded as participants completed two sets of 478 trials, with each set showing different emotional primes: angry and neutral face–voice primes or happy and neutral face–voice primes, followed by either a touch or no-touch stimulus on a viewed hand while the participant's own corresponding hand was touched. Set order was counterbalanced across participants. Within each set, 432 trials were experimental trials, and an additional 46 trials were randomly chosen from a set of experimental and catch trials. Across experimental trials, an equal number of emotional and neutral primes, and of touch and no-touch images, were presented. For catch trials, the touch image was displayed twice, enveloping a 100 ms presentation of the neutral image (a double touch). Only the second of the touch images was accompanied by a felt touch. Participants were asked to count the number of such viewed double touches. Feedback on counting performance was provided every 48 trials. Catch trials were inserted to ensure that attention was paid to the images. These trials were excluded from somatosensory ERP analyses.

In each trial, the face–voice prime was presented for 400 ms, followed by the neutral hand stimulus for 600 ms. The neutral hand stimulus was then replaced by either a touch or no-touch stimulus for 200 ms before returning to the neutral hand stimulus (600 ms), giving the impression of apparent motion of the pencil to touch the hand (touch) or the space next to it (no-touch). Suprathreshold mechanical tactile stimuli were presented during the touch or no-touch image (a 200 ms tap to either left or right hand). At the end of each trial there was a blank inter-trial interval of between 300 and 600 ms (figure 1*b*). All trial types within each set were randomly intermixed.

### (d) E lectroencephalography recording and event-related potential analysis

EEG was recorded from 64 EasyCap scalp electrodes (Easycap, Herrsching, Germany), referenced off-line to the average of all scalp electrodes. Online EEG was amplified, band-pass filtered at 0.01–100 Hz and digitized at 1000 Hz. Offline EEG was filtered with a low-pass filter of 30 Hz, and epoched from 100 ms before to 400 ms after face–voice prime stimulus onset (visual ERPs), and from 100 ms before to 400 ms after tactile stimulus onset (somatosensory ERPs). Trials with eye blinks and other artefacts (a voltage exceeding ±100 µV at any electrode relative to the 100 ms pre-stimulus baseline) measured in this interval were excluded from analysis of visual and somatosensory ERPs. After averaging over trials in each condition of emotional set (happy versus angry) and emotional prime (emotional versus neutral), mean visual ERP amplitudes covering the N170 component (145–185 ms after stimulus onset) were extracted from twelve occipitotemporal electrodes (PO3, PO4, PO5, PO6, PO7, PO8, P5, P6, P7, P8, O1 and O2). After averaging over trials in each condition of emotional set (happy versus angry), emotional prime (emotional versus neutral), and viewed touch (touch versus no-touch image), mean somatosensory ERP amplitudes were extracted from a measurement window covering early somatosensory components P45 through P100 (40–110 ms) for nine lateral electrodes over contralateral central and parietal somatosensory cortex (C2, C4, C6, CP2, CP4, CP6, P2, P4 and P6 for left-hand touch; C1, C3, C5, CP1, CP3, CP5, P1, P3 and P5 for right-hand touch). Time windows and sites were chosen based on visual inspection of the presence of visual and somatosensory components and peak voltages in grand averaged ERPs collapsed over all experimental conditions.

## 3. Results

### (a) Survey scores

Scores of all surveys and their subscales of interest were averaged and compared between groups (table 1). As expected, CDS and OPD-SQ scores differed markedly between groups. Higher OPD-SQ scores, previously associated with dissociation [57], indicated greater disturbances in self–other differentiation in the high-DP group compared with the low-DP group. This novel finding suggests that self–other confusion also typifies those with frequent symptoms of DP-DR. Interoceptive awareness as measured by MAIA was comparable across groups, except for the ‘not distracting’ dimension, suggesting that those with higher levels of depersonalization are less able to ignore or distract themselves from sensations of pain or discomfort (see also [64], for related findings on altered selective attention in clinical depersonalization).

Higher scores on the PHQ9 and STICSA subscales in the high- compared with the low-DP group indicated that the groups differed not just in dissociative symptoms but also in depression and anxiety. While comorbid depression and anxiety are typical in clinical DP-DR (e.g. [33,65]), it does mean that group differences in survey measures and ERPs may be at least partially due to differences in depression and anxiety or poorer mental health in general.

### (b) Visual event-related potentials: N170

After collapsing mean voltages from all left and all right occipitotemporal electrodes, respectively, a mixed ANOVA with the within-subject factors emotional set (happy versus angry), emotional prime (emotional versus neutral) and hemisphere (left versus right), and the between-subject factor group (low-DP versus high-DP), tested the effects of DP-DR symptoms on the structural encoding of others' emotions (N170 component) (see electronic supplementary material, table S1 for mean voltages in each condition).

There was a significant main effect of emotional prime, *F*_1,48_ = 28.97, *p* < 0.001, *ηp*^2^ = 0.376), demonstrating larger N170 mean amplitudes in response to emotional than neutral face–voice stimuli. Emotional priming effects differed between groups (emotional prime × group: *F*_1,48_ = 7.58, *p* = 0.008, *ηp*^2^ = 0.14) and between emotional sets (emotional prime × emotional set: *F*_1,48_ = 7.87, *p* = 0.007, *ηp*^2^ = 0.14), but not between hemispheres or for any combination of these factors (*F*_1,48_ ≤ 3.61, *p* ≥ 0.064, *ηp*^2^ ≤ 0.07). Pairwise comparisons of the estimated marginal means for emotional and neutral primes at each level of emotional set and at each level of group showed larger effects of emotional priming in angry relative to happy sets, and in low-DP relative to high-DP participants.

**Figure 2.**
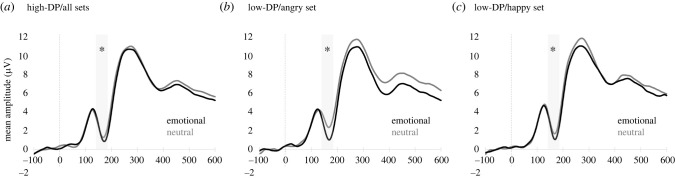
Visual event-related potentials (ERPs) of emotional priming effects (emotional versus neutral face–voice stimuli) found in analyses: for high-depersonalization (high-DP) group across all sets (pooled data, *a*), low-DP group in the angry set (pooled data, *b*), low-DP group in the happy set (pooled data, *c*). Shaded areas show time window used for analyses encompassing component N170. Asterisks indicate significant differences between emotional and neutral waveforms.

**Figure 3.**
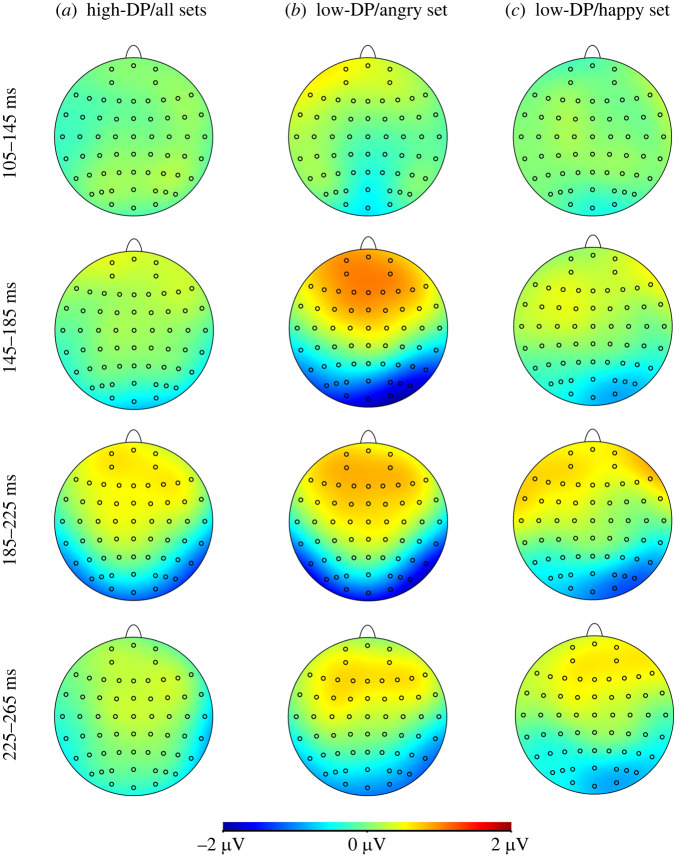
Temporal evolution of topographic maps over four consecutive time windows covering components P1 (105–145 ms), N170 (145–185 ms) and P2 (ascending flank) (185–225 ms, 225 –265 ms) for the voltage differences between emotional and neutral face–voice conditions found in analyses: for high-depersonalization (high-DP) group across all sets (*a*), low-DP group in the angry set (*b*), low-DP group in the happy set (*c*).

Separate follow-up ANOVAs were run for each group. The high-DP group showed a significant main effect of emotional prime, *F*_1,28_ = 8.45, *p* = 0.007, *ηp*^2^ =0.23, which was not modified by emotional set, hemisphere or their combinations, *F* ≤ 2.68, *p* ≥ 0.113, *ηp*^2^ ≤ 0.09. However, the low-DP group's main effect of emotional prime, *F*_1,20_ = 16.61, *p* = 0.001, *ηp*^2^ = 0.45, differed between emotional sets, *F*_1,20_ = 4.44, *p* = 0.048, *ηp*^2^ = 0.18. No other interactions with emotional prime were significant (*F* ≤ 3.72, *p* ≥ 0.068, *ηp*^2^ ≤ 0.16). Pairwise comparisons at each level of emotional set showed that effects of emotional priming were much larger in the angry set, *F*_1,28_ = 4.80, *p* = 0.001, *ηp*^2^ = 0.40, than in the happy set, *F*_1,20_ = 5.38, *p* = 0.020, *ηp*^2^ = 0.24, but present for both (figure 2 for visual ERPs and figure 3 for temporal evolutions of topographic maps confirming activation differences at occipitotemporal sites that peaked at latencies corresponding to the N170 component).

In sum, we found evidence for enhanced processing of emotional others compared with neutral others at face-structural encoding stages, which was particularly pronounced for negative (angry) relative to positive (happy) emotional expressions, but overall much weaker in people with more severe symptoms of DP-DR. A lack of emotional differentiation in this group, relative to those with low levels of symptoms, suggests that DP-DR is associated with altered processing of others' emotions.

### (c) Somatosensory event-related potentials: P45 through P100

After collapsing mean voltages from central and parietal somatosensory electrodes, a mixed ANOVA for the within-subject factors emotional set (happy versus angry), emotional prime (emotional versus neutral), viewed touch (touch versus no-touch image) and touched hand (left versus right), and the between-subject factor group (low- versus high-DP), tested the effects of DP-DR symptoms and emotional priming on mirror touch (touch versus no-touch differences at P45–P100) (see electronic supplementary material, table S2 for mean voltages in each condition).

There was a significant main effect of viewed touch, *F*_1,48_ = 6.23, *p* = 0.016, *ηp*^2^ = 0.12, indicating enhanced positivities at central and parietal sites during touch viewing versus no-touch viewing (mirror touch). There was also a significant four-way interaction between emotional set, emotional prime, viewed touch and group, *F*_1,48_ = 5.85, *p* = 0.019, *ηp*^2^ = 0.11. No other interaction with viewed touch was significant (*F* ≤ 3.35, *p* ≥ 0.074, *ηp*^2^ ≤ 0.07). Pairwise comparisons of the estimated marginal means for viewed touch and no-touch at each level of emotional set, emotional prime and group showed that the largest effects of viewed touch were seen following angry face–voice primes for the high-DP group while the smallest effects of viewed touch were seen following angry face–voice primes for the low-DP group.

**Figure 4.**
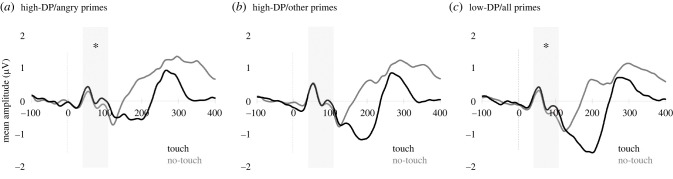
Somatosensory event-related potentials (ERPs) of mirror-touch effects (touch versus no-touch) found in analyses for: high-depersonalization (high-DP) group following angry primes (*a*), high-DP group following other primes (pooled data, *b*), low-DP group following all primes (pooled data, *c*). Shaded areas show time window used for analyses encompassing components P45 through P100. Asterisks indicate significant differences between touch and no-touch waveforms.

Separate follow-up ANOVAs for each group revealed that the high-DP group lacked a main effect of viewed touch, *F*_1,28_ < 1, *p* = 0.328, *ηp*^2^ = 0.03, but showed an interaction between emotional set, emotional prime and viewed touch, *F*_1,28_ = 7.17, *p* = 0.012, *ηp*^2^ = 0.20. Pairwise comparisons at each level of emotional set and emotional prime showed that effects of viewed touch were largest and significant following angry face–voice primes, *F*_1,28_ = 4.80, *p* = 0.037, *ηp*^2^ = 0.15, but not present in any other condition, *F*_1,28_ ≤ 3.27, *p* ≥ 0.081, *ηp*^2^ ≤ 0.11. By contrast, the low-DP group showed a significant main effect of viewed touch, *F*_1,20_ = 5.38, *p* = 0.031, *ηp*^2^ = 0.21, which did not interact with emotional set, emotional prime, touched hand or their combinations, *F* ≤ 1.47, *p* ≥ 0.239, *ηp*^2^ ≤ 0.07 (figure 4).

In sum, we found that emotional context modified effects of mirror touch only in those with more severe symptoms of depersonalization. Specifically, angry others were the only emotional stimuli associated with subsequent early cortical resonance with viewed tactile stimuli in those with higher levels of symptoms. Embodiment of tactile stimuli in people with lower levels of symptoms was unaffected by others’ emotions, however.

### (d) Correlational tests

We further tested whether significant effects of emotional prime and mirror touch following angry stimuli systematically related to survey measures of interest (i.e. those found to differ between groups, table 1) across the total sample. It was expected that more severe mental health symptoms on survey measures would be associated with less emotional N170 enhancement for angry face–voice primes and with more mirror touch following such angry primes. Emotional priming effects were calculated by collapsing mean N170 amplitudes in the angry set across hemispheres and subtracting amplitudes in response to emotional (angry) primes from those to neutral primes. Mirror-touch effects were calculated by collapsing mean P45–P100 amplitudes following angry primes across touched hand and subtracting amplitudes in trials where no-touch was viewed from those where touch was viewed. The resulting two variables were not normally distributed and therefore entered into non-parametric one-tailed correlations with scores from CDS (total), CDS (anomalous body experience), CDS (emotional numbing), STICSA (cognitive anxiety), PHQ9, OPD-SQ (self–other confusion) and MAIA (not distracting).

**Figure 5.**
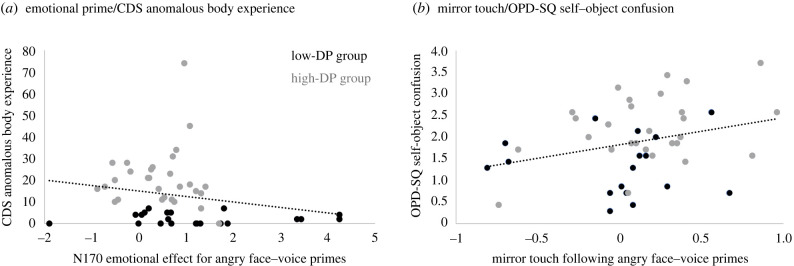
Scatterplots illustrating the relationships between event-related potential (ERP) effects and survey measures. (*a*) Smaller effects of emotional prime (N170 to angry versus neutral face–voice stimuli) relate to higher reports of anomalous body experiences. (*b*) Larger effects of mirror touch (touch versus no-touch viewing on early somatosensory ERPs) following angry face–voice primes relate to higher reports of self–other confusion.

We found that the angry emotional prime effect moderately negatively correlated with CDS (anomalous body experience), Spearman's rho (*ρ*)= −0.30, *p* = 0.016. In other words, less emotional differentiation between angry and neutral primes was seen in those with more symptoms of disembodiment (figure 5). Mirror touch weakly positively correlated with OPD-SQ (self–other confusion *ρ*= 0.25, *p* = 0.046, showing that there were larger effects of cortical resonance following angry primes in those reporting more self–other confusions (figure 5). No other correlations were significant (*ρ* ≤ 0.21, *p* ≥ 0.072).

## 4. Discussion

The present study was designed to test the electrophysiological correlates of emotional face perception and how being confronted with others' emotions may modify the mirroring of subsequent touch in people who have high or low levels of depersonalization–derealization (DP-DR) symptoms.

Similar to previous studies, we found that for all participants the structural encoding of faces was affected by emotions: there were higher visual N170 amplitudes in response to emotional (happy, angry) face–voice stimuli relative to neutral face–voice stimuli (see [49] for a review and meta-analysis). N170 stages of processing are thought to encompass the encoding of facial structure in the occipital face area of the ventral occipitotemporal cortex (e.g. [48,50]), but they have also been linked with the encoding of facial identity (e.g. [66]) and emotional expression (e.g. [49,52]). Similar to our results, previous studies have found the largest emotional modulations of N170 for angry faces, followed by fearful or happy faces, without particular lateralization of these effects [49,52].

We also found evidence of mirror touch: higher somatosensory P45 and P100 amplitudes in response to the observation of touch relative to no-touch stimuli [11,12,14,43]. Somatosensory ERP components P45 and P100 arise from processing in S1 [67–72], although the origins of P100 have variably been placed in posterior S1 (e.g. [70]) and bilateral S2 (e.g. [73]). This suggests that somatosensory resonance with others’ bodily sensations takes place at early, implicit levels of processing, in line with previous studies showing mirror touch at these ERP components ([11,12,14,37,43]; for review see [74]). More importantly, we showed that both of these visual and somatosensory processes differed between low- and high-DP groups. Here we will describe each group in turn.

For the low-DP group, emotional modulations of N170 face processing were particularly pronounced for negative (angry) relative to positive (happy) emotional expressions, similar to previous research (see [49,52]). The subsequent somatosensory cortical resonance with observed touch was not modulated by the emotion context of face–voice primes, however. That is, the extent of mirroring others' bodily sensations was unaffected by prior confrontation with an emotional (positive or negative versus neutral) other.

Bodily resonance is assumed to be the basis of more complex social phenomena like emotional contagion and affective empathy (e.g. [19,20]). While this mirroring process occurs implicitly and automatically, here we find that it may not, however, be automatically modified by the influence of preceding emotional contexts. On the one hand, this suggests that mirroring is a relatively stable process that is robust to affective influences. On the other hand, it is of course also possible that tactile mirroring may prove to be more malleable in situations with different, stronger, more dynamic or realistic emotional stimuli, or when there is more subjective personal involvement with the emotional other. Indeed, previous studies have shown that the self-relatedness of visual stimuli on which touch is observed (e.g. own versus stranger's face; human versus rubber hand) increases tactile mirroring responses [11,12,38,39,44,45]. Previous studies have also shown that the emotional enhancement of mirror touch in healthy participants may be restricted to fear [23,24].

Compared with the low-DP group, the high-DP group showed both weaker N170 emotional differentiation of face–voice primes and altered P45–P100 somatosensory resonance following those primes. While in low-DP observers there were stronger emotional modulations of N170 face processing for negative (angry) relative to positive (happy) emotional expressions, the high-DP group showed smaller N170 differences between emotional and neutral conditions overall, with no modulation by positive versus negative emotional contexts. This weaker N170 emotional modulation is in line with studies showing altered processing of others' emotions in those with DP-DR [53]. In Lemche *et al*.'s study [53], patients exhibited reduced limbic (hypothalamus, amygdala) responses to increasingly intense (happy and sad) facial emotions, coupled with increases in dorsal prefrontal activity, suggesting that emotions were suppressed in DP-DR patients. These results were related to the emotional numbing typically reported by those with DP-DR (see also [75]).

DP-DR has been thought of as reflecting a protective mechanism against the experience of threatening emotions (e.g. intense anxiety; [76–78]). The findings from the present study may help to further elucidate this protective mechanism. In correlational analyses across all participants, we found that N170 responses to angry versus neutral primes were weaker in those with more anomalous body experiences. Anomalous body experiences such as feelings of disembodiment were significantly more typical of our high- than our low-DP group, as shown in direct group comparisons of this CDS subcategory. Our findings thus suggest a link between the emotional suppression experienced in DP-DR and feelings of disembodiment.

For the mirroring of subsequent touch, we found that those with more symptoms of DP-DR showed somatosensory resonance only in the context of preceding angry face–voice primes. This is in contrast to the low-DP group, who showed somatosensory resonance regardless of prime condition. Such group differences support previous suggestions that people with more frequent or stronger symptoms of DP-DR have altered somatosensory resonance compared with those with fewer such symptoms [11,13]. Previous studies showed that the mirroring of touch on one's own face, which was observed in a low-DP group, was much weaker in a high-DP group [11] and that reduced self-face bias in tactile mirroring was predicted by higher DP-DR symptom scores [13].

It may therefore be that lower reactivity to others' (especially negative) emotions, which may indicate suppression of negative emotions [53], is related to feelings of disembodiment and reduced somatosensory resonance. The idea that emotional processes are strongly intertwined with somatosensory ones is in line with sensorimotor simulation accounts of emotion recognition (e.g. [79,80]). In the present study, however, we showed that prior confrontation with negative emotional (angry) others can increase cortical somatosensory resonance in high-DP groups. In addition, correlational analyses across all participants showed that tactile mirroring following angry primes was larger in those reporting more self–other confusion, as measured with the OPD-SQ. In other words, experiencing oneself as less psychologically separate from others was related to greater effects from others’ negative emotions on somatosensory resonance with subsequent stimuli. Direct comparison between high- and low-DP groups showed that self–other confusion was significantly more characteristic of our high- than our low-DP group. This finding adds to reports linking dissociative symptoms in borderline personality disorder with overarching subscales of the OPD-SQ [57], and is the first study to our knowledge to show that self–other confusion specifically relates to DP-DR symptoms, as well as to effects on somatosensory resonance following confrontation with others' negative emotions. While the former conclusion was statistically well supported, the latter must be taken with some caution, however, as the relationship between self–other confusion and somatosensory resonance was only weak in our sample of participants. Nevertheless, it suggests intriguing possibilities for further research into self–other confusion as a potentially transdiagnostic symptom of dissociative disorders (e.g. [21]).

In the OPD-SQ, self–other confusion is indicated by items describing psychological experiences such as ‘Sometimes I'm afraid that the boundary between me and others will disappear’. However, self–other confusion may also entail bodily experiences. For example, previous studies have found that high-DP participants are more susceptible to experiencing illusory embodiment of others, such as in the rubber-hand illusion [35] or in mirror-pain synaesthesia [81], both of which have been theorized to be based on insufficient self–other distinction (e.g. [82]).

Put together, it may thus be speculated that self–other confusion may protect against feelings of separateness, helplessness and loneliness, but can also engender feelings of threat, especially when the other is felt to be aggressive. Interestingly, we found that those who reported greater disconnection with their bodily self (anomalous body experiences) had weaker N170 responses to angry versus neutral primes, which may index reduced emotional processing. Although this relationship was only moderate and must therefore remain somewhat speculative, it suggests that disconnecting from one's self and body may be a defence mechanism that reduces the threatening nature of highly negative feelings like fear and anger, which are engendered by heightened resonance with others in conditions of greater self–other confusion. In addition, disconnecting from one's bodily self also reduces perception of one's own feelings, because their embodied aspects are experienced less strongly. This mechanism may relate to the mechanism underlying the subjective emotional numbness reported by patients with DP-DR (e.g. [75]). While emotional numbing serves a self-protective function in situations of high stress or trauma, post-traumatic models of dissociative disorders like DP-DR posit that patients experience a persistent state of threat and, therefore, of emotional numbness (e.g. [76–78]). The role of self–other confusion in relation to DP-DR symptoms like emotional numbness should be explored in more depth in future studies.

Furthermore, we propose that future studies could investigate mirroring processes and their modulation through confrontation with emotional others in more realistic contexts. It is conceivable that the presentation of emotional others via computer screen, rather than in-person, only weakly activated the cortical networks related to face processing and social interactions (see [83]). Future studies should therefore address the role of intensity and self-relatedness in emotional primes and their effect on subsequent somatosensory resonance in person rather than digitally. In addition to improving the realism of emotional stimuli, the presence of a dynamic other who is touched may also enhance touch simulation in observers. This may be particularly so if the observed touch is delivered interpersonally rather than through mechanical means.

In conclusion, this is the first study to our knowledge to measure somatosensory resonance in emotional contexts in people with high and low levels of DP-DR symptoms. We show evidence of weaker emotional processing, altered resonance with subsequent somatosensory stimuli, and how they relate with self-reported feelings of disembodiment and self–other confusion, respectively. Overall, our findings suggest that disconnecting from one's body and self (core symptom of DP-DR) may be a defence mechanism to protect from the threat of negative feelings, which may be exacerbated through self–other confusion. This defence mechanism may also underlie symptoms of emotional numbness.

## Data Availability

Anonymized data are available at https://osf.io/6wmbg/ [84]. Supplementary material is available online [85].

## References

[1] Gallese V, Sinigaglia C. 2010 The bodily self as power for action. Neuropsychologia **48**, 746-755. (10.1016/j.neuropsychologia.2009.09.038) 19835895

[2] Legrand D. 2006 The bodily self: the sensori-motor roots of pre-reflective self-consciousness. Phenomenol. Cogn. Sci. **5**, 89-118. (10.1007/s11097-005-9015-6)

[3] Rochat P, Striano T. 2000 Perceived self in infancy. Infant Behav. Dev. **23**, 513-530. (10.1016/S0163-6383(01)00055-8)

[4] Filippetti ML, Johnson MH, Lloyd-Fox S, Dragovic D, Farroni T. 2013 Body perception in newborns. Curr. Biol. **23**, 2413-2416. (10.1016/j.cub.2013.10.017) 24268410 PMC3898688

[5] Zmyj N, Jank J, Schutz-Bosbach S, Daum MM. 2011 Detection of visual–tactile contingency in the first year after birth. Cognition **120**, 82-89. (10.1016/j.cognition.2011.03.001) 21458785

[6] Stern N. 1985 The interpersonal world of the infant: a view from psychoanalysis and developmental psychology. London, UK: Karnac Books.

[7] Winnicott DW. 1960 Ego distortion in terms of true and false self. In The maturational processes and the facilitating environment (ed. DW Winnicott), pp. 140-152. Madison, CT: International Universities Press.

[8] Fonagy P, Gergely G, Jurist EL (eds). 2004 Affect regulation, mentalization and the development of the self. London, UK: Karnac Books.

[9] Porciello G, Bufalari I, Minio-Paluello I, Di Pace E, Aglioti SM. 2018 The ‘enfacement’ illusion: a window on the plasticity of the self. Cortex **104**, 261-275. (10.1016/j.cortex.2018.01.007) 29478669

[10] Schettler A, Raja V, Anderson ML. 2019 The embodiment of objects: review, analysis, and future directions. Front. Neurosci. **13**, 1332. (10.3389/fnins.2019.01332) 31920499 PMC6923672

[11] Adler J, Schabinger N, Michal M, Beutel ME, Gillmeister H. 2016 Is that me in the mirror? Depersonalisation modulates tactile mirroring mechanisms. Neuropsychologia **85**, 148-158. (10.1016/j.neuropsychologia.2016.03.009) 26970140

[12] Adler J, Gillmeister H. 2019 Bodily self-relatedness in vicarious touch is reflected at early cortical processing stages. Psychophysiology **56**, e13465. (10.1111/psyp.13465) 31464351

[13] Farmer H, Cataldo A, Adel N, Wignall E, Gallese V, Deroy O, Hamilton A, Ciaunica A. 2020 The detached self: investigating the effect of depersonalisation on self-bias in the visual remapping of touch. Multisens. Res. **34**, 365-386. (10.1163/22134808-bja10038) 33535167

[14] Rigato S, Bremner AJ, Gillmeister H, Banissy MJ. 2019 Interpersonal representations of touch in somatosensory cortex are modulated by perspective. Biol. Psychol. **146**, 107719. (10.1016/j.biopsycho.2019.107719) 31207259

[15] Bouquet CA, Shipley TF, Capa RL, Marshall PJ. 2011 Motor contagion. Exp. Psychol. **58**, 71-78. (10.1027/1618-3169/a000069) 20494864

[16] Chartrand TL, Bargh JA. 1999 The chameleon effect: the perception–behavior link and social interaction. J. Pers. Social Psychol. **76**, 893. (10.1037/0022-3514.76.6.893) 10402679

[17] Spengler S, Brass M, Kühn S, Schütz-Bosbach S. 2010 Minimizing motor mimicry by myself: self-focus enhances online action-control mechanisms during motor contagion. Conscious. Cogn. **19**, 98-106. (10.1016/j.concog.2009.12.014) 20116291

[18] Lakin JL, Jefferis VE, Cheng CM, Chartrand TL. 2003 The chameleon effect as social glue: evidence for the evolutionary significance of nonconscious mimicry. J. Nonverb. Behav. **27**, 145-162. (10.1023/A:1025389814290)

[19] Pineda JA, Moore AR, Elfenbeinand H, Cox R. 2009 Hierarchically organized mirroring processes in social cognition: the functional neuroanatomy of empathy. In *Mirror neuron systems: the role of mirroring processes in social cognition* (ed. JA Pineda), pp. 135–160. New York, NY: Humana Press. (10.1007/978-1-59745-479-7)

[20] Rizzolatti G, Fabbri-Destro M. 2008 The mirror system and its role in social cognition. Curr. Opin. Neurobiol. **18**, 179-184. (10.1016/j.conb.2008.08.001) 18706501

[21] Eddy CM. 2022 The transdiagnostic relevance of self-other distinction to psychiatry spans emotional, cognitive and motor domains. Front. Psychiat. **13**, 797952. (10.3389/fpsyt.2022.797952) PMC8960177 35360118

[22] Prochazkova E, Kret ME. 2017 Connecting minds and sharing emotions through mimicry: a neurocognitive model of emotional contagion. Neurosci. Biobehav. Rev. **80**, 99-114. (10.1016/j.neubiorev.2017.05.013) 28506927

[23] Cardini F, Bertini C, Serino A, Ladavas E. 2012 Emotional modulation of visual remapping of touch. Emotion **12**, 980. (10.1037/a0027351) 22390704

[24] Scarpazza C, di Pellegrino G, Ladavas E. 2014. Emotional modulation of touch in alexithymia. *Emotion* **14**, 602. (10.1037/a0035888) 24708501

[25] Michal M, Beutel ME, Jordan J, Zimmermann M, Wolters S, Heidenreich T. 2007 Depersonalization, mindfulness, and childhood trauma. J. Nerv. Ment. Dis. **195**, 693-696. (10.1097/NMD.0b013e31811f4492) 17700303

[26] Sierra M, David AS. 2011 Depersonalization: a selective impairment of self-awareness. Conscious. Cogn. **20**, 99-108. (10.1016/j.concog.2010.10.018) 21087873

[27] Simeon D. 2004 Depersonalisation disorder - a contemporary overview. CNS Drugs **18**, 343-354. (10.2165/00023210-200418060-00002) 15089102

[28] Trueman D. 1984 Depersonalization in a nonclinical population. J. Psychol. **116**, 107-112. (10.1080/00223980.1984.9923624) 6607991

[29] Spiegel D, Loewenstein RJ, Lewis‐Fernández R, Sar V, Simeon D, Vermetten E, Dell PF. 2011 Dissociative disorders in DSM‐5. *Depress. Anxiety* **28**, E17–E45. (10.1002/da.20923) 22134959

[30] American Psychiatric Association. 2013 Diagnostic and statistical manual of mental disorders, 5th edn. Arlington, VA: APA.

[31] Hunter EC, Sierra M, David AS. 2004 The epidemiology of depersonalisation and derealisation. A systematic review. Social Psychiatry Psychiatr. Epidemiol. **39**, 9-18. (10.1007/s00127-004-0701-4) 15022041

[32] Lee WE, Kwok CH, Hunter EC, Richards M, David AS. 2012 Prevalence and childhood antecedents of depersonalization syndrome in a UK birth cohort. Social Psychiatry Psychiatr. Epidemiol. **47**, 253-261. (10.1007/s00127-010-0327-7) PMC3355298 21181112

[33] Yang J, Millman LM, David AS, Hunter EC. 2023 The prevalence of depersonalization-derealization disorder: a systematic review. J. Trauma Dissociation **24**, 8-41. (10.1080/15299732.2022.2079796) 35699456

[34] Ketay S, Hamilton HK, Haas BW, Simeon D. 2014 Face processing in depersonalization: an fMRI study of the unfamiliar self. Psychiatry Res. **222**, 107-110. (10.1016/j.pscychresns.2014.02.003) 24582597 PMC5510159

[35] Kanayama N, Sato A, Ohira H. 2009 The role of gamma band oscillations and synchrony on rubber hand illusion and crossmodal integration. Brain Cogn. **69**, 19-29. (10.1016/j.bandc.2008.05.001) 18555572

[36] Banissy MJ, Kadosh RC, Maus GW, Walsh V, Ward J. 2009. Prevalence, characteristics and a neurocognitive model of mirror-touch synaesthesia. *Exp. Brain Res.* **198**, 261–272. (10.1007/s00221-009-1810-9) 19412699

[37] Bufalari I, Aprile T, Avenanti A, Di Russo F, Aglioti SM. 2007 Empathy for pain and touch in the human somatosensory cortex. Cereb. Cortex **17**, 2553-2561. (10.1093/cercor/bhl161) 17205974

[38] Cardini F, Costantini M, Galati G, Romani GL, Làdavas E, Serino A. 2011 Viewing one's own face being touched modulates tactile perception: an fMRI study. J. Cogn. Neurosci. **23**, 503-513. (10.1162/jocn.2010.21484) 20350177

[39] Cardini F, Tajadura-Jiménez A, Serino A, Tsakiris M. 2013 It feels like it's me: interpersonal multisensory stimulation enhances visual remapping of touch from other to self. J. Exp. Psychol. Hum. Percept. Perform. **39**, 630-637. (10.1037/a0031049) 23276110 PMC3750640

[40] Deschrijver E, Wiersema JR, Brass M. 2015 The interaction between felt touch and tactile consequences of observed actions: an action-based somatosensory congruency paradigm. Social Cogn. Affective Neurosci. **11**, 1162-1172. (10.1093/scan/nsv081) PMC4927036 26152577

[41] Deschrijver E, Wiersema JR, Brass M. 2017 Action-based touch observation in adults with high functioning autism: can compromised self-other distinction abilities link social and sensory everyday problems? Social Cogn. Affect. Neurosci. **12**, 273-282. (10.1093/scan/nsw126) PMC5390705 27613781

[42] Gillmeister H. 2014 A new perceptual paradigm to investigate the visual remapping of others’ tactile sensations onto one’s own body shows “mirror touch” for the hands. Front. Psychol. **5**, 95. (10.3389/fpsyg.2014.00095) PMC3918665 24575070

[43] Martínez-Jauand M, Gonzalez‐Roldan AM, Munoz MA, Sitges C, Cifre I, Montoya P. 2012. Somatosensory activity modulation during observation of other's pain and touch. *Brain Res.* **1467**, 48–55. (doi:10.1016/j.brain res.2012.05.055) 10.1016/j.brainres.2012.05.055 22683688

[44] Serino A, Pizzoferrato F, Làdavas E. 2008 Viewing a face (especially one's own face) being touched enhances tactile perception on the face. Psychol. Sci. **19**, 434-438. (10.1111/j.1467-9280.2008.02105.x) 18466402

[45] Serino A, Giovagnoli G, Làdavas E. 2009 I feel what you feel if you are similar to me. Plos One **4**, e4930. (10.1371/journal.pone.0004930) 19293933 PMC2654168

[46] Michal M, Wiltink J, Subic-Wrana C, Zwerenz R, Tuin I, Lichy M, Brähler E, Beutel ME. 2009 Prevalence, correlates, and predictors of depersonalization experiences in the German general population. J. Nerv. Ment. Dis. **197**, 499-506. (10.1097/NMD.0b013e3181aacd94) 19597357

[47] Simeon D, Guralnik O, Schmeidler J, Sirof B, Knutelska M. 2001 The role of childhood interpersonal trauma in depersonalization disorder. Am. J. Psychiatry **158**, 1027-1033. (10.1176/appi.ajp.158.7.1027) 11431223

[48] Eimer M. 2011 The face-sensitive N170 component of the event-related brain potential. In *The Oxford handbook of face perception* (eds A Calder, G Rhodes, M Johnson, J Haxby), 329–344. Oxford, UK: Oxford University Press.

[49] Hinojosa JA, Mercado F, Carretié L. 2015 N170 sensitivity to facial expression: a meta-analysis. Neurosci. Biobehav. Rev. **55**, 498-509. (10.1016/j.neubiorev.2015.06.002) 26067902

[50] Rossion B. 2014 Understanding face perception by means of human electrophysiology. Trends Cogn. Sci. **18**, 310-318. (10.1016/j.tics.2014.02.013) 24703600

[51] Hogendoorn H, Kammers M, Haggard P, Verstraten F. 2015 Self-touch modulates the somatosensory evoked P100. Exp. Brain Res. **233**, 2845-2858. (10.1007/s00221-015-4355-0) 26105753 PMC4575392

[52] Schindler S, Bublatzky F. 2020 Attention and emotion: an integrative review of emotional face processing as a function of attention. Cortex **130**, 362-386. (10.1016/j.cortex.2020.06.010) 32745728

[53] Lemche E, Surguladze SA, Giampietro VP, Anilkumar A, Brammer MJ, Sierra M, Phillips ML. 2007 Limbic and prefrontal responses to facial emotion expressions in depersonalization. Neuroreport **18**, 473-477. (10.1097/WNR.0b013e328057deb3) 17496806

[54] Sierra M, Berrios GE. 2000 The Cambridge Depersonalisation Scale: a new instrument for the measurement of depersonalisation. Psychiatry Res. **93**, 153-164. (10.1016/S0165-1781(00)00100-1) 10725532

[55] Sierra M, Baker D, Medford N, David AS. 2005 Unpacking the depersonalization syndrome: an exploratory factor analysis on the Cambridge depersonalization scale. Psychol. Med. **35**, 1523-1532. (10.1017/S0033291705005325) 16164776

[56] Ehrenthal JC, Dinger U, Horsch L, Komo-Lang M, Klinkerfuß M, Grande T, Schauenburg H. 2012 Der OPD-Strukturfragebogen (OPD-SF): erste Ergebnisse zu Reliabilität und Validität [The OPD Structure Questionnaire (OPD-SQ): first results on reliability and validity]. Psychother. Psychosom. Med. Psychol. **62**, 25-32. (10.1055/s-0031-1295481) [In German with English abstract.] 22271173

[57] Sole S. 2014 Dissociative symptoms and the quality of structural integration in borderline personality disorder. D.Clin.Psy thesis, University College London. See https://discovery.ucl.ac.uk/id/eprint/1448837.

[58] Kessler RC, Gruber M, Hettema JM, Hwang I, Sampson N, Yonkers KA. 2008 Co-morbid major depression and generalized anxiety disorders in the National Comorbidity Survey follow-up. Psychol. Med. **38**, 365-374. (10.1017/S0033291707002012) 18047766 PMC2745899

[59] Kroenke K, Spitzer RL, Williams JB. 2001 The PHQ-9: validity of a brief depression severity measure. J. Gen. Intern. Med. **16**, 606-613. (10.1046/j.1525-1497.2001.016009606.x) 11556941 PMC1495268

[60] Grös DF, Antony MM, Simms LJ, McCabe RE. 2007 Psychometric properties of the State-Trait Inventory for Cognitive and Somatic Anxiety (STICSA): comparison to the State-Trait Anxiety Inventory (STAI). Psychol. Assess. **19**, 369-381. (10.1037/1040-3590.19.4.369) 18085930

[61] Mehling WE, Price C, Daubenmier JJ, Acree M, Bartmess E, Stewart A. 2012 The Multidimensional Assessment of Interoceptive Awareness (MAIA). PloS One **7**, e48230. (10.1371/journal.pone.0048230) 23133619 PMC3486814

[62] Tottenham N et al. 2009 The NimStim set of facial expressions: judgments from untrained research participants. Psychiatry Res. **168**, 242-249. (10.1016/j.psychres.2008.05.006) 19564050 PMC3474329

[63] Belin P, Fillion-Bilodeau S, Gosselin F. 2008 The Montreal Affective Voices: a validated set of nonverbal affect bursts for research on auditory affective processing. Behav. Res. Methods **40**, 531-539. (10.3758/BRM.40.2.531) 18522064

[64] Schabinger N, Gillmeister H, Berti S, Michal M, Beutel ME, Adler J 2018. Detached and distracted: ERP correlates of altered attentional function in depersonalisation. *Biol. Psychol.* **134**, 64–71. (10.1016/j.biopsycho.2018.02.014) 29486234

[65] Michal M et al. 2016 A case series of 223 patients with depersonalization-derealization syndrome. BMC Psychiatry **16**, 203. (10.1186/s12888-016-0908-4) 27349226 PMC4924239

[66] Keyes H, Brady N, Reilly RB, Foxe JJ. 2010 My face or yours? Event-related potential correlates of self-face processing. Brain Cogn. **72**, 244-254. (10.1016/j.bandc.2009.09.006) 19854553

[67] Allison T, McCarthy G, Wood CC, Jones SJ. 1991 Potentials evoked in human and monkey cerebral cortex by stimulation of the median nerve: a review of scalp and intracranial recordings. Brain **114**, 2465-2503. (10.1093/brain/114.6.2465) 1782527

[68] Allison T, McCarthy G, Wood CC. 1992 The relationship between human long-latency somatosensory evoked potentials recorded from the cortical surface and from the scalp. Electroencephalogr. Clin. Neurophysiol. **84**, 301-314. (10.1016/0168-5597(92)90082-M) 1377999

[69] Cardini F, Longo MR. 2016 Congruency of body-related information induces somatosensory reorganization. Neuropsychologia **84**, 213-221. (10.1016/j.neuropsychologia.2016.02.013) 26902158

[70] Schaefer M, Xu B, Flor H, Cohen LG. 2009 Effects of different viewing perspectives on somatosensory activations during observation of touch. Hum. Brain Mapp. **30**, 2722-2730. (10.1002/hbm.20701) 19172650 PMC6870795

[71] Schubert R, Blankenburg F, Lemm S, Villringer A, Curio G. 2006 Now you feel it—now you don't: ERP correlates of somatosensory awareness. Psychophysiology **43**, 31-40. (10.1111/j.1469-8986.2006.00379.x) 16629683

[72] Schubert R, Ritter P, Wüstenberg T, Preuschhof C, Curio G, Sommer W, Villringer A. 2008 Spatial attention related SEP amplitude modulations covary with BOLD signal in S1—a simultaneous EEG–fMRI study. Cereb. Cortex **18**, 2686-2700. (10.1093/cercor/bhn029) 18372293

[73] Zhu Z, Disbrow EA, Zumer JM, McGonigle DJ, Nagarajan SS. 2007 Spatiotemporal integration of tactile information in human somatosensory cortex. BMC Neurosci. **8**, 21. (10.1186/1471-2202-8-21) 17359544 PMC1838913

[74] Salami A, Andreu-Perez J, Gillmeister H. 2020 Symptoms of depersonalisation/derealisation disorder as measured by brain electrical activity: a systematic review. Neurosci. Biobehav. Rev. **118**, 524-537. (10.1016/j.neubiorev.2020.08.011) 32846163

[75] Phillips ML, Sierra M. 2003 Depersonalization disorder: a functional neuroanatomical perspective. Stress **6**, 157-165. (10.1080/1025389031000138538) 13129809

[76] Hunter ECM, Phillips ML, Chalder T, Sierra M, David AS. 2003 Depersonalisation disorder: a cognitive–behavioural conceptualisation. Behav. Res. Ther. **41**, 1451-1467. (10.1016/S0005-7967(03)00066-4) 14583413

[77] Sierra M. 2008 Depersonalization disorder: pharmacological approaches. Expert Rev. Neurother. **8**, 19-26. (10.1586/14737175.8.1.19) 18088198

[78] Stein DJ, Simeon D. 2009 Cognitive-affective neuroscience of depersonalization. CNS Spectr. **14**, 467-471. (10.1017/S109285290002352X) 19890227

[79] Adolphs R, Damasio H, Tranel D, Cooper G, Damasio AR. 2000. A role for somatosensory cortices in the visual recognition of emotion as revealed by three-dimensional lesion mapping. *J. Neurosci.* **20**, 2683–2690. (10.1523/JNEUROSCI.20-07-02683.2000) PMC6772225 10729349

[80] Wood A, Rychlowska M, Korb S, Niedenthal P. 2016 Fashioning the face: sensorimotor simulation contributes to facial expression recognition. *Trends Cogn. Sci.* **20**, 227-240. (10.1016/j.tics.2015.12.010) 26876363

[81] Bowling NC, Botan V, Santiesteban I, Ward J, Banissy MJ. 2019 Atypical bodily self-awareness in vicarious pain responders. Phil. Trans. R. Soc. B **374**, 20180361. (10.1098/rstb.2018.0361) 31630646 PMC6834008

[82] Santiesteban I, Bird G, Tew O, Cioffi MC, Banissy MJ. 2015 Mirror-touch synaesthesia: difficulties inhibiting the other. Cortex **71**, 116-121. (10.1016/j.cortex.2015.06.019) 26188789

[83] Zhao N, Zhang X, Noah JA, Tiede M, Hirsch J. 2023 Separable Processes for Live ‘In-Person’ and Live ‘Zoom-like’ Faces. Imaging Neuroscience **1**, 1-17. 10.1162/imag_a_00027 PMC12007548 40799691

[84] Gillmeister H, Šmate I, Savva D, Li H, Parapadakis C, Adler J. 2024 Confrontation with others' emotions changes bodily resonance differently in those with low and high levels of depersonalisation. OSF. (https://osf.io/6wmbg/) 10.1098/rstb.2023.0248 PMC11444244 39005042

[85] Gillmeister H, Šmate I, Savva D, Li H, Parapadakis C, Adler J. 2024 Confrontation with others' emotions changes bodily resonance differently in those with low and high levels of depersonalisation. Figshare. (10.6084/m9.figshare.c.7308148) PMC11444244 39005042

